# Transcranial Doppler Ultrasound: A Review of the Physical Principles and Major Applications in Critical Care

**DOI:** 10.1155/2013/629378

**Published:** 2013-12-12

**Authors:** Jawad Naqvi, Kok Hooi Yap, Gulraiz Ahmad, Jonathan Ghosh

**Affiliations:** ^1^University Hospital South Manchester, Southmoor Road, Wythenshawe, Manchester M23 9LT, UK; ^2^Manchester Royal Infirmary, Oxford Road, Manchester M13 9WL, UK; ^3^Royal Oldham Hospital, Rochdale Road, Manchester OL1 2JH, UK

## Abstract

Transcranial Doppler (TCD) is a noninvasive ultrasound (US) study used to measure cerebral blood flow velocity (CBF-V) in the major intracranial arteries. It involves use of low-frequency (≤2 MHz) US waves to insonate the basal cerebral arteries through relatively thin bone windows. TCD allows dynamic monitoring of CBF-V and vessel pulsatility, with a high temporal resolution. It is relatively inexpensive, repeatable, and portable. However, the performance of TCD is highly operator dependent and can be difficult, with approximately 10–20% of patients having inadequate transtemporal acoustic windows. Current applications of TCD include vasospasm in sickle cell disease, subarachnoid haemorrhage (SAH), and intra- and extracranial arterial stenosis and occlusion. TCD is also used in brain stem death, head injury, raised intracranial pressure (ICP), intraoperative monitoring, cerebral microembolism, and autoregulatory testing.

## 1. Introduction

Transcranial Doppler (TCD), first described in 1982 [1], is a noninvasive ultrasound (US) study that involves the use of a low-frequency (≤2 MHz) transducer probe to insonate the basal cerebral arteries through relatively thin bone windows. TCD allows dynamic monitoring of cerebral blood flow velocity (CBF-V) and vessel pulsatility over extended time periods with a high temporal resolution. It is relatively inexpensive, repeatable, and its portability offers increased convenience over other imaging methods, allowing continuous bedside monitoring of CBF-V, which is particularly useful in the intensive care setting [2]. The technique is however highly operator dependent, which can significantly limit its utility [3–6]. It also has a long learning curve to acquire the three-dimensional understanding of cerebrovascular anatomy necessary for competency [3]. Furthermore, approximately 10–20% of patients have inadequate transtemporal acoustic windows [2, 4, 7].

Current applications of TCD in adults and children include vasospasm in sickle cell disease [8], subarachnoid haemorrhage (SAH) [9], intra- and extracranial arterial stenosis and occlusion [10, 11], brain stem death [12], head injury, raised intracranial pressure (ICP) [13], intraoperative monitoring [14], impaired vasomotor function [15], and cerebral microembolism in right to left cardiac shunts [16]. TCD has also been widely used to investigate cerebral pressure autoregulation [17]. Combined with waveform morphology, indices derived from flow velocity readings such as Gosling's pulsatility index (PI) and the Lindegaard ratio (LR) allow identification of increased cerebrovascular resistance, vasospasm, and hyperdynamic flow states, which characterise the above clinical conditions.

This paper will review the underlying physical principles of TCD, flow indices frequently used in clinical care, and critical care indications for TCD in adults and children (discussion of neonatal TCD applications is beyond the scope of this paper).

## 2. Methods

A MEDLINE search performed by the authors in March 2013 of “Transcranial Doppler Ultrasound” in all fields yielded 7134 results. A further search combined with the descriptor AND ““acute care” OR “critical care” OR “intensive care” OR “neuro-critical care”” yielded 514 results. Filtering for English language review articles retrieved 72 articles. Eleven articles focusing on critical care applications of TCD in adults published in the last 10 years were retrieved [2, 4, 5, 9, 18–24]. Abstracts were screened to deem final appropriateness before the article and its references were consulted in depth to gather information for this current review.

## 3. Review

### 3.1. Physical Principles

The Doppler effect states that where a sound wave strikes a moving object, such as an erythrocyte, the reflected wave undergoes a change in frequency (the Doppler shift *f*_*d*_) directly proportional to the velocity (*v*) of the reflector. The following equation derived from this principle is the basis for calculating CBF-V with TCD:

(1) $$\begin{array}{c} v=\frac{\left(c\times f_{d}\right)}{2\times f_{0}\times \cos\;\theta }, \end{array}$$

where *c* is the speed of the incident wave, *f*_0_ is the incident pulse frequency, and *θ* is the angle of the reflector relative to the US probe [25].

**Figure 1:**
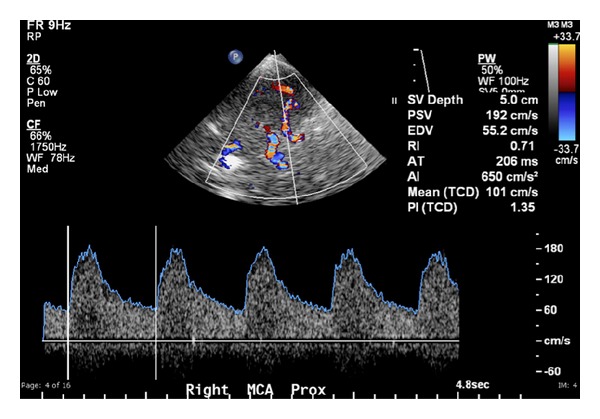
Right MCA TCD waveform (bottom) with colour Doppler (top).

TCD relies on pulsed wave Doppler to image vessels at various depths [3]. Received echoes generate an electrical impulse in the US probe and are processed to calculate *f*_*d*_ and *v*, to produce a spectral waveform with peak systolic velocity (PSV) and end diastolic velocity (EDV) values (see Figure 1).

**Figure 2:**
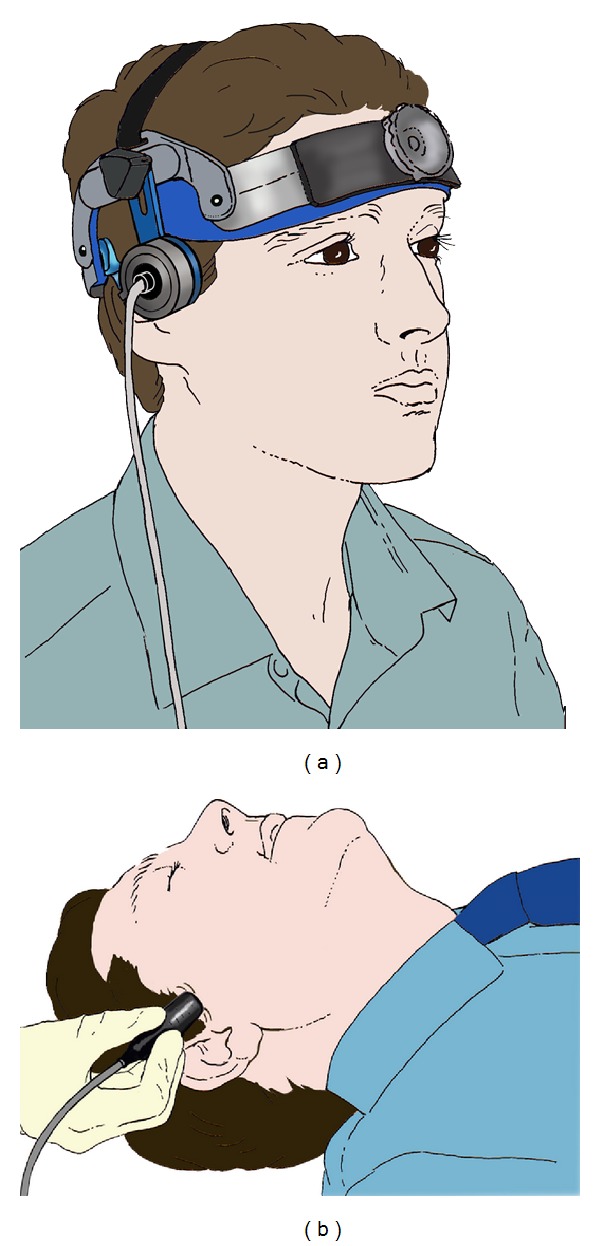
TCD headset and TCD handheld probe applied over the transtemporal window.  Figure 2(b) is adapted from Nicoletto and Burkman [3]. Permission obtained. The copyright owner for the original image from which Figure 2(b) is adapted, is ASET (American Society of Electroneurodiagnostic Technologists), the Neurodiagnostic Society.

An ultrasound (US) frequency of ≤2 MHz is required to penetrate the skull and reach the intracranial vasculature. Depending on procedure duration, the US probe is fixed in a headset or manually applied (see Figures 2(a) and 2(b)).

Acoustic windows are skull regions, either foramina or thin bone, that transmit US waves to the basal cerebral circulation [3]. There are four acoustic windows, namely, the transtemporal, suboccipital (transforaminal), transorbital, and submandibular (retromandibular). The transtemporal window, located above the zygomatic ridge between the lateral canthus of the eye and auricular pinna, is most frequently used and can insonate the middle (MCA), anterior (ACA), posterior cerebral arteries (PCA), and terminal internal carotid artery (ICA) [2, 3]. However, between 10% and 20% of patients have inadequate transtemporal windows [2, 4, 7].

The target artery is insonated by selecting an appropriate acoustic window, probe angle, and sample volume depth [3]. The artery is recognized through flow direction, resistance (pulsatility), and velocity in addition to waveform changes induced by dynamic manoeuvres such as proximal carotid artery compression and tapping over bony landmarks [2, 3].  Table 1 provides a summary of the insonation characteristics of the cerebral vasculature. Procedural techniques for tracing each artery are described elsewhere [2, 3].

### 3.2. TCD Indices

Mean flow velocity (MFV) is a central parameter in TCD and is equal to (PSV + (EDV × 2))/3 [3]. A number of physiological factors may influence MFV, as described in Table 2.

When MFV is increased, it may indicate stenosis, vasospasm, or hyperdynamic flow. A decreased value may indicate hypotension, decreased CBF, ICP, or brain stem death [18]. Focal arterial stenosis or vasospasm is represented by an increased MFV within a 5–10 mm segment, usually by >30 cm/s compared with the asymptomatic side [26].

Gosling's pulsatility index (PI) provides information on downstream cerebral vascular resistance and is equal to (PSV-EDV)/MFV [27]. PI is normally 0.5 to 1.19 [27]. Proximal stenosis or occlusion may lower the PI below 0.5 due to downstream arteriolar vasodilation whilst distal occlusion or constriction may increase the PI above 1.19 [26]. A PI less than 0.5 may also indicate an arteriovenous malformation as vessel resistance in proximal vessels is reduced due to continuous distal venous flow [28]. PI positively correlates with ICP; a PI change of 2.4% is reflected by a 1 mmHg change in ICP [29].

The Pourcelot resistivity index (RI) is equal to (PSV-EDV)/PSV with values >0.8 indicating increased downstream resistance. Derangements of RI reflect similar disease patterns as observed with an abnormal PI [18].

The Lindegaard ratio (LR) allows differentiation between hyperdynamic flow and vasospasm and is defined as MCA MFV/extracranial ICA MFV [30]. In the context of a high MFV, an LR <3 indicates hyperdynamic flow and >3 indicates vasospasm [31]. A modified LR (BA MFV/average of left and right extracranial VA MFV) and Sloan's hemispheric ratio (ACA MFV/ECICA MFV) can be similarly applied to the BA and ACA, respectively (see [5] for a summary of threshold values). MFV and LR measurements used to grade vasospasm severity are presented in Table 3 [31, 32].

### 3.3. Specialist Indices

Vasodilatory stimulation via breath holding and CO_2_-induced hypercapnia can detect an impaired cerebral vasomotor reserve (VMR) and impending stroke [15].

The breath-holding index (BHI) is equal to ((CBF-V max − CBF-V min)/time of breath hold) × 100 [33]. A BHI >0.6 is normal; between 0.21 and 0.60 is impaired VMR, whilst ≤0.20 is significantly impaired VMR [34].

The CO_2_ challenge VMR index is calculated using the average CBF-V at baseline, during hypercapnia and hypocapnia, and is equal to (Hypercapnia CBF-V − Hypocapnia CBF-V)/(Baseline CBF-V) × 100. A value greater than 70% is normal, 39–69% is mild to moderately reduced VMR, 16–38% is severely reduced VMR, and ≤15% is exhausted VMR [34].

Microembolic signal (MES) detection is useful in intra-operative monitoring, grading right to left shunts; and identifying patients with internal carotid stenosis who may benefit from endarterectomy [35–37]. Basic identification criteria for MES include [38] the following:

1. transient character (typically <300 ms), but duration is dependent on passage time through the sample volume;
2. high intensity (amplitude >3 dB above background—appears bright);
3. typically unidirectional and random appearance in the cardiac cycle;
4. audible as “snaps, tonal chirps, or moans” [38].

### 3.4. Applications

Following a MEDLINE search, as described in Section 2, a wide range of TCD indications were identified, which are summarised in Table 4. The indications are subdivided into ischaemic cerebrovascular disease, periprocedural and neurointensive care categories as per the American Academy of Neurology [39].

Our discussion will focus on the main applications of TCD in critical care highlighted by our literature search including vasospasm in sickle cell disease, SAH, acute stroke, brain stem death, traumatic brain injury (TBI), raised ICP, cardiac shunts, and autoregulatory testing. (Discussion of peri-procedural TCD applications, including the evaluation of extracranial carotid disease [11, 36, 42], intracranial stenosis [6, 43–45] and monitoring in carotid endarterectomy [14, 35, 46–51] and other neurovascular [52–55] and cardiac procedures [56–58] are beyond the scope of this paper and the reader is directed to the referenced articles.)

### 3.5. Sickle Cell Disease

Patients with sickle cell disease are at risk from a spectrum of brain injuries that include subclinical infarction, acute stroke and haemorrhage; the prevalence of acute stroke in sickle cell disease is 600 per 100,000 patient-years [59]. The underlying pathology involves distal ICA, proximal MCA and ACA stenosis, and occlusion as a result of an increasing circulation of irreversibly sickled cells and their adherence to the vascular endothelium.

CBF-V >200 cm/s in asymptomatic children with sickle cell disease is associated with an increased risk of stroke of 10,000 per 100,000 patient-years [60]. Treatment with blood transfusion in such children can reduce the risk of stroke by >90% [61]. Therefore, TCD screening of children between 2- and 6-years old is recommended on a 6–12 monthly basis, involving measurement of the time-averaged mean maximum CBF-V in bilateral MCA, bifurcation, distal ICA, ACA, PCA, and BA [8]. Patients with a time averaged mean maximum CBF-V in all arteries of <170 cm/sec are deemed normal [8]. If a value >200 cm/s in any artery is observed, then blood transfusion is recommended to reduce sickle haemoglobin to less than 30% of total haemoglobin and prevent stroke [8, 59].

### 3.6. Subarachnoid Haemorrhage

The delayed vasospasm of the cerebral vasculature is angiographically proven in up to 70% of cases of SAH and usually occurs 4 to 17 days after haemorrhage [9, 62]. It has significant implications on mortality and morbidity with approximately 25% of SAH patients developing delayed ischemic deficits due to vasospasm [4, 18, 40, 62]. The pathogenesis is unclear but is thought to involve the breakdown of blood in the subarachnoid space and secondary cellular mechanisms which culminate in vasoconstriction of adjacent intracranial arteries [9, 63].

Angiography is the gold standard for detecting vasospasm but is an invasive technique and unsuited to dynamic monitoring [2, 41]. TCD, however, is non-invasive, portable, and able to dynamically assess vasospasm and monitor the effectiveness of intervention including triple-H therapy (hypertension, haemodilution, and hypervolaemia), transluminal balloon angioplasty, or pharmacologic vasodilation [9]. Additionally, TCD is a prognostic indicator and can guide initiation of triple H-therapy [2, 4]. Conventionally, serial TCD measurements are performed daily after SAH. Table 2 outlines the flow criteria used to grade vasospasm severity on TCD.

**Figure 3:**
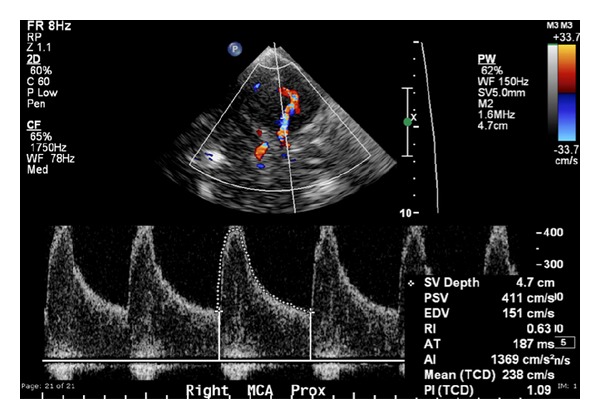
A 70-year-old woman with SAH. TCD demonstrates an increased PSV and MFV in the right MCA, consistent with severe vasospasm.

TCD identifies MCA and BA vasospasm with a high sensitivity and specificity [39]. A systematic review of 26 studies comparing TCD with angiography found that MCA MFV >120 cm/s was 99% specific and 67% sensitive to angiographic vasospasm of ≥25% [64]. In a retrospective study of 101 patients, MCA MFV >120 cm/s was 72% specific and 88% sensitive for ≥33% angiographic vasospasm with a negative predictive value (NPV) of 94% for MFV <120 cm/s [65]. In the same study, MFV >200 cm/s was 98% specific and 27% sensitive with a positive predictive value (PPV) of 87% for angiographic vasospasm of ≥33% [65]. Therefore, MFV <120 cm/s and >200 cm/s may accurately predict absence and presence of angiographic MCA, vasospasm, respectively (Figure 3). The LR theoretically allows differentiation from hyperdynamic flow; however, its usefulness is limited as it fails to improve upon the identification of MCA vasospasm or development of delayed cerebral ischaemia (DCI) [20].

For the detection of >50% BA vasospasm, by using concomitant thresholds of MFV >85 cm/s and modified LR >3, TCD has a sensitivity of 92% and specificity of 97% [32]. Specificity may rise to 100% with MFV >95 cm/s [66]. Additionally, the modified LR has a strong correlation with BA diameter, shown to be >3 in 100% of patients with >50% vasospasm in one study [32, 67].

However, for vasospasm of the ACA and PCA sensitivity of TCD is notably inferior [39]. In a cohort of 57 patients after SAH who underwent TCD within 24 hours of angiography ACA MFV ≥120 cm/s was 18% sensitive and 65% specific for vasospasm and PCA MFV ≥90 cm/s was 48% sensitive and 69% specific for vasospasm [68].

Despite the high sensitivity that may be achieved for MCA and BA vasospasm, the prognostic ability of TCD and potential to improve outcome in SAH are challenged [9, 18]. In a cohort of 580 SAH patients, only 84% of those with delayed cerebral ischaemia (DCI) had evidence of angiographic vasospasm [69]. Furthermore, DCI, and not vasospasm, was significantly associated with adverse outcome [69]. This may be due to additional pathogenic mechanisms such as reperfusion injury, hydrocephalus, and a disrupted blood-brain barrier contributing to neurological decline [20]. However, rate of MFV increase may predict DCI with a rise in MFV of >20% or >65 cm/s per day increase in MFV between days 3 and 7 predictive of poor outcome [4].

To summarise, TCD is useful for the identification of MCA and BA vasospasm in SAH; however, evidence for its prognostic value is limited. The American Heart Association (AHA) has accordingly recommended TCD as a reasonable tool to monitor for development of vasospasm in their evidence-based guidance on the management of SAH [70].

### 3.7. Acute Ischaemic Stroke: Diagnosis and Prognosis

#### 3.7.1. Diagnosis

TCD is a convenient, low-cost, and rapidly repeatable test compared to MR and CT in suspected ischaemic stroke [5, 71]. However, as with stenoocclusive disease, high sensitivity and specificity are demonstrated only in the proximal anterior circulation. In a cohort of 48 patients with angiographic proven occlusion TCD had an overall sensitivity of 83% and specificity of 94%, with sensitivity optimal in the proximal ICA (94%) and MCA (93%), and significantly less in the terminal VA (56%) and BA (60%) [72].

#### 3.7.2. Prognosis

The temporal resolution of TCD is a particular advantage over other techniques. By performing serial TCD examinations, haemodynamic changes following ischaemic stroke that would otherwise go undetected by a single MRA can be elicited [71]. Such haemodynamic changes have the potential to predict clinical outcome.

Haemodynamic changes before and after intravenous tissue plasminogen activator (tPA) administration in ischaemic stroke are classified by the thrombolysis in brain ischaemia (TIBI) grading system [73]. Residual flow is graded as either 0: absent, 1: minimal, 2: blunted, 3: dampened, 4: stenotic, or 5: normal [73]. TIBI grade and TIBI grade improvement are correlated with stroke severity, mortality, and clinical recovery based on the National Institutes of Health Stroke Scale (NIHSS) and modified Rankin Score (mRS) [4, 73–76].

A meta-analysis has shown that recanalization observed on TCD within 6 hours of symptom onset is significantly associated with clinical improvement at 48 hours (OR 4.31, 95% CI: 2.67–6.97) and functional independence at 3 months (OR 6.75, 95% CI 3.47–13.12) [77]. To add to this, an abrupt increase in TIBI grade or stepwise increase over 30 minutes indicates more complete recanalisation and is significantly associated with better short-term outcome on the NIHSS, compared with recanalisation taking more than 30 minutes [75]. Mortality is significantly increased in MCA occlusion versus MCA patency on admission treated without thrombolysis (odds ratio 2.46 95% CI: 1.33–4.52) and also in persisting MCA occlusion at two hours after tPA bolus [76, 77].

In addition, using the TIBI grading system TCD can detect early (<2 hours) reocclusion (flow decrease ≥1 TIBI grade) following tPA which may occur in up to 34% patients with initial recanalization [76]. Early re-occlusion is associated with a significantly poorer outcome at 3 months and a higher in-hospital mortality compared to sustained recanalization [76].

Aside from TIBI grading, the site and severity of occlusion observed on TCD may help predict outcome. In a study of 335 patients with acute stroke who received tPa and underwent TCD, distal MCA occlusions had the greatest chance of early recanalisation at 44%, compared with 30% in the proximal MCA, 30% in the BA, and <10% in the terminal ICA [21]. However, an unknown number of patients were excluded from this study due to inadequate acoustic windows, and very few posterior circulation occlusions were present in the sample. In the multicenter Neurosonology in Acute Ischaemic Stroke (NAIS) trial, the extent of MCA occlusion observed on TCD was significantly associated with functional outcome at 3 months [78]. Out of those with a patent MCA, 71% had a good functional outcome whereas of those with a main stem occlusion, 88% were dead or functionally dependent at 3 months after stroke [78].

#### 3.7.3. Treatment

Discussion of the treatment of acute ischemic stroke with TCD is beyond the scope of this paper and the reader is directed to the following dedicated review articles [6, 79–83].

In conclusion, TCD is highly sensitive and specific (>80%) for ICA and MCA occlusion [72, 74]. By monitoring recanalisation via TIBI grading, TCD is also a reliable prognostic indicator in MCA occlusive stroke [73, 75, 76]. However, CTA and MRA are preferable as firstline imaging techniques in ischaemic stroke due to the operator dependence of TCD and poor ability to access the posterior circulation [6].

### 3.8. Brain Stem Death

Brain stem death is usually diagnosed by clinical examination and extended observation [84]. Confirmatory tests such as EEG can be employed to facilitate a rapid diagnosis in cases where organ preservation is needed in preparation for possible transplant surgery [19, 84]. However, brain stem injury, paralysis, pharmacological sedation with barbiturates, or hypothermia may prevent diagnosis based on clinical examination and EEG [19]. TCD is an alternative confirmatory test in such scenarios.

**Figure 4:**
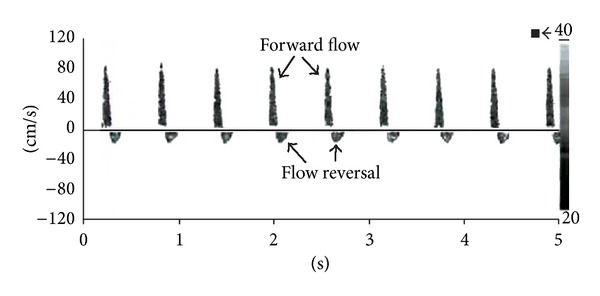
The TCD waveform in raised ICP or brain stem death. This oscillating MCA waveform demonstrates antegrade systolic flow with retrograde diastolic flow, consistent with raised ICP or brain stem death. Reproduced from Nicoletto and Burkman [26]. Permission obtained. Copyright owner ASET (American Society of Electroneurodiagnostic Technologists), the Neurodiagnostic Society.

**Figure 5:**
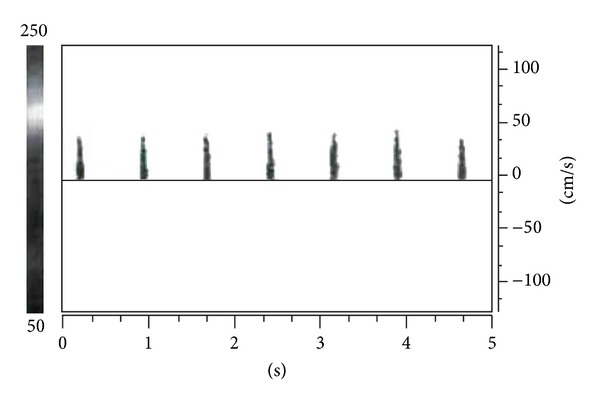
The TCD waveform in raised ICP or brain stem death. This MCA waveform demonstrates absent diastolic flow and small systolic spikes consistent with the late stages of raised ICP or brain stem death. Reproduced from Nicoletto and Burkman [26]. Permission obtained. Copyright owner ASET (American Society of Electroneurodiagnostic Technologists), the Neurodiagnostic Society.

Criteria for the diagnosis of cerebral circulatory arrest (which precedes brain stem death) on TCD state that one of the following waveforms must be observed in the BA, bilateral ICA, and bilateral MCA on two examinations at least 30 minutes apart [12]:

1. an oscillating waveform (equal systolic forward flow and diastolic reversed flow, i.e., zero net flow; see Figure 4), or
2. small systolic spikes of <200 ms duration and <50 cm/s PSV with no diastolic flow (see Figure 5), or
3. disappearance of intracranial flow with typical signals observed in the extracranial circulation.

There are reports of TCD demonstrating a 100% agreement with the gold standard of arteriography for confirmation of brainstem death [85]. A meta-analysis and a technology assessment by the American Academy of Neurology have however shown that sensitivity and specificity range between 89% and 100% and 97% and 100%, respectively [19, 39]. Due to a certain proportion of patients having an inadequate acoustic window, the sensitivity is unlikely to ever reach 100%, but sensitivity and specificity may improve by repeated testing, which is a practical possibility given the noninvasiveness of TCD [84, 85].

As noted previously, TCD is an operator-dependent technique. It requires significant prior experience as well as knowledge of the underlying physiology of brain stem death and the diagnostic criteria to derive firm conclusions on the presence of cerebral circulatory arrest [19].

### 3.9. Traumatic Brain Injury and Raised Intracranial Pressure

Traumatic brain injury (TBI) may lead to hypoperfusion (day 0), hyperaemia (days 1–3), vasospasm (days 4–15), and raised ICP [86]. TCD can noninvasively identify such complications (see TCD indices above) and provide prognostic information [18, 39].

Previous work with invasive ^133^Xe clearance methods has shown that the extent of hypoperfusion in the acute setting after TBI correlates with outcome at 6 months based on the Glasgow Outcome Scale (GOS) [87]. TCD can avoid use of invasive CBF measurement techniques and provide similar prognostic information. A low-flow velocity state defined as an MCA MFV of <35 cm/s within 72 hours of head injury has been shown to predict unfavourable outcome at 6 months (GOS score 1–3: death, vegetative state, or severe disability) with an odds ratio of 3.9 (CI 1.2–13) [88]. However, on multivariate analysis, this association was significantly less (OR 1.2 CI: 0.25–5.9), with initial GCS being a stronger predictor of outcome.

The severity of vasospasm may also predict outcome on the GOS; in a study of 116 SAH patients, moderate BA vasospasm (MFV >60 cm/s) was associated with permanent neurological deficit, and severe BA vasospasm (MFV >85 cm/s) was associated with vegetative state (*P* = 0.00019) [89]. However, no relationship between the severity of MCA vasospasm and clinical outcome was demonstrated [89]. In a separate study of 50 patients with head injury who underwent TCD insonation of the MCA, ACA, and BA in the first 7 days after TBI, significantly more patients in the vasospasm and hyperaemia groups experienced a poor outcome at 6 months (GOS 1–3) compared to those without any significant flow velocity change [90]. The highest MFV recorded, independent of vasospasm or hyperaemia, was also predictive of outcome with those in the poor outcome group (GOS 1–3) having a significantly greater highest MFV [90].

On TCD, raised ICP exhibits a sequential waveform, beginning with an increased PI and decreased MFV and EDV, followed by zero diastolic flow and criteria 1–3 listed in Section 3.8 [91]. A significant correlation between PI and ICP (correlation coefficient 0.938 *P* < 0.0001) was demonstrated in a group of 81 patients who underwent TCD MCA PI measurements combined with invasive ICP measurements [92]. A regression line was derived as ICP = (11.1 × PI) − 1.43, which could determine an ICP via the PI within ±4.2 mmHg of the actual ICP, which is reasonably accurate. Using this regression line, an ICP of >20 mmHg could also be determined with 89% sensitivity and 92% specificity [92]. Furthermore, in a study of 125 patients with severe TBI, poor outcomes (GOS 1–3) were associated with a significant rise in MCA PI (1.56 versus 1, *P* < 0.0001) within 24 hours of injury [13]. Additionally, a PI ≥1.56 predicted 83% of patients who had a poor outcome at 6 months, whereas a PI ≤1 identified 71% of patients with a good outcome (GOS 4–5) [13].

As mentioned above TCD can noninvasively estimate absolute ICP and CCP, avoiding the complications of invasive monitoring [2, 93]. However, there are various formulae proposed for this purpose, which demonstrate unacceptably wide confidence intervals and remain to be fully validated [2, 18, 93]. Hence, at present, TCD is reserved for assessing change, rather than absolute CPP, in TBI [2].

In summary, TCD can identify after-TBI haemodynamic changes, which can be used as early predictors of outcome at 6 months based on the GOS with a moderate degree of reliability. Noninvasive TCD estimates of ICP and CCP require further validation.

### 3.10. Cardiac Shunts

Paradoxical embolism through right to left cardiopulmonary shunts (e.g., patent foramen ovale) is an important cause of stroke in those under 55 years of age [94].

TCD offers a noninvasive method to assess and classify the grade of shunting via an MES grading scheme, which can also help stratify patients according to risk of stroke (Table 5) [95, 96]. A peripheral injection of agitated saline or Echovist (Schering AG, Germany; a microparticle contrast agent) is administered and the patient is asked to perform a Valsalva manoeuvre, with the TCD probe place over the MCA [95]. The number of microembolic signals (MES) observed up to 40 seconds after the end of the injection are counted [95].

Earlier reviews identify a sensitivity of approximately 70–100% for right-to-left shunts using TCD compared to the gold standard of transesophgeal ultrasound (TEU) [39, 98]. However, in a more recent study of 321 simultaneous TEU and TCD experiments, TCD detected right-to-left shunts with a sensitivity of only 38% and specificity of 99% compared to TEU [37]. TCD performance was better for detection of large PFOs (>30 microbubbles detected by TEU in the left atrium) with a sensitivity of 100% and specificity of 92.5% [37].

Transesophageal ultrasonography (TEU), although more invasive, holds further advantages over TCD as it can localise the shunt and identify presence of an atrial septal aneurysm, another risk factor for stroke in the young [20, 39, 94]. Therefore, TEU remains the first line tool in assessment of RLS where the patient is able to tolerate an invasive approach.

### 3.11. Cerebral Autoregulation

Cerebral pressure autoregulation refers to the maintenance of CBF despite changes in CPP between 50 and 150 mmHg [99]. An impairment of this autoregulatory response has been demonstrated in TBI [100], stroke [101], carotid disease [102], and more controversially syncope [103]. Impaired autoregulation may be of use in prognosticating such patients and determining treatment strategies [17].

Lassen first described the cerebral autoregulatory curve by collating the results of separate studies, which measured CBF using indicator dilution techniques under steady state conditions [99]. Indeed, the majority of initial research into cerebrovascular autoregulation focused on adopting a steady state (or static) approach to measuring CBF following a pharmacologic stimulus to alter CPP [17]. However, with the advent of TCD the time course of CBF changes following a pressure stimulus, using CBF-V as a surrogate marker could be dynamically monitored. This had the advantage of minimising the effect of confounding factors such as changes in PaCO_2_ and autonomic activity that may feature in CBF measurements taken hours apart under steady state conditions [17, 104].

TCD combined with thigh cuff deflation was pioneered by Aaslid in 1989 [105], and this has been followed by a variety of other nonpharmacologic methods to evoke the pressure response including carotid artery compression (transient hyperaemic response) [106], valsalva manoeuvres [107], head up-tilt [108], and lower body negative pressure [103, 109]. Such mechanical methods avoid the direct autoregulatory effects of pharmacologic pressure stimuli used more extensively in the past [18, 103, 110].

Despite the ability of TCD to observe a dynamic autoregulatory response, a large number of TCD studies adopt a static model to autoregulatory testing in patients [103]. In this context, the static autoregulatory index (sARI) or static rate of regulation (sROR), defined as the % change in CVR/% change in CPP, has been used [111]. This represents a useful tool to classify autoregulation ranging from 0, an absent response, to 1, a fully responsive autoregulatory system. Static methods however require pharmacologic or mechanical step changes in CPP, which may be inappropriate and unsafe in critically unwell patients [17, 101, 112]. The significant time interval between CBF-V measurements can also potentiate the effect of confounding factors, which shift the autoregulatory curve, producing misleading results [104]. Furthermore, there is a failure to capture the evolution and latency of the autoregulatory response [111].

In the arena of dynamic testing, no gold standard index exists [113]. The Mx index defines the degree of correlation between CPP and MFV; a positive correlation indicates pressure-dependent blood flow and loss of autoregulation whereas an absent correlation is a sign of an intact autoregulatory system [112, 114]. A limitation of this index is that correlation may be significant but the slope negligible [17]. The dynamic autoregulatory index (dARI) initially proposed by Tiecks et al. involves fitting the observed CBF-V response, following a pressure stimulus, to one of 10 theoretical CBF-V response curves, which model absent autoregulation (curve 0) through to fully intact autoregulation (curve 9) [111].

The use of mechanical nonpharmacologic stimuli can however induce significant changes in PaCO_2_ and cerebral metabolic activity, which confound CBF [103, 115]. Hence, use of spontaneous fluctuations in CPP secondary to low-frequency respiratory waves to dynamically ascertain the presence of autoregulation has been proposed as an ideal method, which overcomes these shortcomings, and is applicable to nearly all patients due to its noninvasiveness [17]. Under this paradigm, not only can the Mx index and dARI be applied within the time domain, but autoregulation can also be determined in the frequency domain by transfer function analysis (TFA) [112]. In TFA, the phase shift between CBF-V and CPP changes is used as a marker of interest [116]. A zero-degree phase shift indicates absence of autoregulation and a negative phase shift (where FV changes before ABP described as a positive phase lead of FV relative to CPP) is presence of autoregulation [116].

In severe head injury impaired autoregulation, determined by the Mx index with use of spontaneous fluctuations of CPP and MFV, is strongly associated with poor outcome at 6 months based on the GOS [114]. Recently, the Sx index, which replaces MFV with SFV, has shown a stronger association than Mx with the GOS [117]. Furthermore, the dARI significantly correlates with the GOS, a threshold of 5.86 conferring a sensitivity of 75% and specificity of 76% for death [118]. Although autoregulation-oriented therapy is advised following these results [114] there is a dearth of prospective trials to evaluate the efficacy of such strategies and hence the Brain Trauma Foundation has advised autoregulatory monitoring as an optional tool in TBI [119].

In ICA stenosis, impaired autoregulation is proposed as a tool to identify patients at highest risk of stroke and thus help optimise selection of surgical candidates [102, 120]. Evidence for this includes the significant decreases in dARI and significant increases in Mx observed ipsilateral to ICA stenoocclusive disease, which correlate with the degree of stenosis [102, 120]. However, significantly abnormal values of dARI and Mx, compared to the control value, were restricted to patients with severe (>80–90%) stenosis, and no clear difference in Mx, Sx, or Dx between asymptomatic and symptomatic patients was demonstrated [102, 120].

In stroke, TCD studies have consistently shown an impairment in ipsilateral cerebral autoregulation and an association with the need for decompressive surgery, neurological decline, and poor outcome [101]. However, the impairment in autoregulation in this population may be as a result of preexisting clinical conditions such as chronic hypertension rather than due to stroke [101].

In the investigation of syncope, the available evidence presents inconsistent conclusions as to whether autoregulatory impairment is a contributory factor [103]. This subset of evidence exemplifies the methodological shortcomings to the TCD assessment of cerebrovascular autoregulation, which limit translation into clinical practice. The wide variety of static and dynamic techniques employed with lack of a gold standard technique and lack of a standardised value to determine impaired autoregulation is critical to preventing the comparability and synthesis of the existing evidence [101, 103, 112]. The failure of studies to assess and control for confounding factors, in particular PaCO_2_, is potentially a major source of error [17, 101, 112]. Furthermore, a large number of studies consist of small patient numbers and are statistically underpowered [103].

The intrinsic technical limitations of TCD further compound the issue. TCD-based studies employ CBF-V as a surrogate measure of CBF. However, CBF-V is only proportional to CBF when vessel cross-sectional area remains constant [121]. Furthermore, since measurements are frequently only taken from the MCA, autoregulatory changes in the posterior circulation may not be realised in addition to specific cortical regional changes, highlighting the limited spatial resolution of TCD [101].

The investigation of cerebral autoregulation using TCD is an area of significant research given the high temporal resolution, noninvasiveness, and convenience of the technique. Significant autoregulatory impairment has been consistently demonstrated after TBI and stroke and is of prognostic importance. In syncope and ICA stenosis, the role of autoregulatory assessment is less clear. Carefully designed studies, which improve the uniformity and reliability of TCD-based cerebral autoregulatory testing across a range of clinical conditions, are warranted [17, 101, 103].

## 4. Conclusions

The portability, repeatability, noninvasiveness, and high temporal resolution of TCD have promoted its use, especially in bedside monitoring of CBF in the critically ill. The majority of supporting evidence pertains to prognostication and initiation of preventative measures in sickle cell disease, SAH, stroke, and TBI.

Further studies linking MES with clinical outcome are warranted in stroke. Carefully designed studies are needed to better determine quality standards in autoregulatory testing and to evaluate the benefit of autoregulation-oriented therapy in TBI.

Invasive techniques appear to remain the gold standard across the majority of clinical applications due to the limited spatial resolution and the assumptions made regarding vessel diameter on TCD. Furthermore, operator dependency is a significant limitation to its clinical utility. However, the temporal resolution and convenience of TCD make it a vital asset to observing the evolution of blood flow changes in the critically ill patient.

## Figures and Tables

**Table 1:** Insonation characteristics of the cerebral vasculature. Adapted from Nicoletto and Burkman [3]. Permission obtained; copyright owner ASET (American Society of Electroneurodiagnostic Technologists), the Neurodiagnostic Society.

Artery	Acoustic window	Probe angle	Depth (mm)	Flow direction	Resistance	Adult MFV (cm/sec)
ECICA	Retromandibular	Superior-medial	45–50	Away	Low	30 ± 9
MCA	Middle transtemporal	Straight/Anterior-superior	30–65	Toward	Low	55 ± 12
ACA	Middle transtemporal	Straight/Anterior-superior	60–75	Away	Low	50 ± 11
PCA—segment 1	Posterior transtemporal	Straight/Posterior	60–70	Toward	Low	39 ± 10
PCA—segment 2	Posterior transtemporal	Straight/Posterior-superior	60–70	Away	Low	40 ± 10
BA	Suboccipital	Superior	80–120	Away	Low	41 ± 10
VA	Suboccipital	Superior lateral	60–75	Away	Low	38 ± 10
OA	Transorbital	Straight	45–55	Toward	High	21 ± 5
Supraclinoid ICA	Transorbital	Superior	65–80	Away	Low	41 ± 11
Parasellar ICA	Transorbital	Inferior	65–80	Toward	Low	47 ± 14

(ECICA: extracranial internal carotid artery, MCA: middle cerebral artery, ACA: anterior cerebral artery, PCA: posterior cerebral artery, BA: basilar artery, OA: ophthalmic artery).

**Table 2:** Factors influencing MFV [18, 20].

Factor	Change in MFV
Age	Increases up to 6–10 years of age then decreases (see [26] for a full range of values)
Sex	Higher MFV in women than men
Pregnancy	Decreased in the 3rd trimester
PCO_2_	Increases with increasing PCO_2_
Mean arterial Pressure (MAP)	Increases with increasing MAP (CBF autoregulates between CPP 50–150 mmHg)
Haematocrit	Increases with decreasing haemotocrit

**Table 3:** Grading of vasospasm severity [31, 32].

Degree of MCA or ICA vasospasm	MFV (cm/s)		LR
Mild (<25%)	120–149	A N D	3–6
Moderate (25–50%)	150–199		3–6
Severe (>50%)	>200		>6

Degree of BA vasospasm	MFV (cm/s)		Modified LR

May represent vasospasm	70–85	A N D	2–2.49
Moderate (25–50%)	>85		2.5–2.99
Severe (>50%)	>85		>3

**Table 4:** TCD applications [2, 4, 18, 39–41]. Categorised as per reference [39].

Ischaemic cerebrovascular disease
Sickle cell disease
Right to left cardiac shunts
Intra and extra-cranial arterial steno-occlusive disease
Arteriovenous malformations and fistulas
Peri-procedural/operative
Cerebral thrombolysis in acute stroke
Carotid endarterectomy
Carotid angioplasty and stenting
Coronary artery bypass surgery
Coronary angioplasty
Prosthetic heart valves
Neurological/Neurosurgical intensive care
Vasospasm after subarachnoid haemorrhage
Raised intracranial pressure
Head injury
Cerebral circulatory arrest and brain death
Intracerebral aneurysm and parenchymal hematoma detection
Others
Pharmacologic vasomotor testing
Cerebral pressure autoregulation
Liver failure/Hepatic encephalopathy
Preeclampsia

**Table 5:** Cardiopulmonary shunt grading based on microembolic signals [95, 97].

Grade of shunt	Number of microembolic signals (MES)
No shunt	0
Low grade shunt	1–10
Medium grade shunt	11–25
High grade shunt	>25 (shower) or uncountable (curtain effect)
